# A Preclinical Evaluation of the Antitumor Activities of Edible and Medicinal Mushrooms: A Molecular Insight

**DOI:** 10.1177/1534735417736861

**Published:** 2017-11-02

**Authors:** Thomson Patrick Joseph, Warren Chanda, Arshad Ahmed Padhiar, Samana Batool, Shao LiQun, MinTao Zhong, Min Huang

**Affiliations:** 1 Dalian Medical University, Dalian, Liaoning, China

**Keywords:** cancer, signaling pathway, genetic alterations, mushroom, molecular insight

## Abstract

Cancer is the leading cause of morbidity and mortality around the globe. For certain types of cancer, chemotherapy drugs have been extensively used for treatment. However, severe side effects and the development of resistance are the drawbacks of these agents. Therefore, development of new agents with no or minimal side effects is of utmost importance. In this regard, natural compounds are well recognized as drugs in several human ailments, including cancer. One class of fungi, “mushrooms,” contains numerous compounds that exhibit interesting biological activities, including antitumor activity. Many researchers, including our own group, are focusing on the anticancer potential of different mushrooms and the underlying molecular mechanism behind their action. The aim of this review is to discuss PI3K/AKT, Wnt-CTNNB1, and NF-κB signaling pathways, the occurrence of genetic alterations in them, the association of these aberrations with different human cancers and how different nodes of these pathways are targeted by various substances of mushroom origin. We have given evidence to propose the therapeutic attributes and possible mode of molecular actions of various mushroom-originated compounds. However, anticancer effects were typically demonstrated in in vitro and in vivo models and very limited number of studies have been conducted in the human population. It is our belief that this review will help the research community in designing concrete preclinical and clinical studies to test the anticancer potential of mushroom-originated compounds on different cancers harboring particular genetic alteration(s).

## Introduction

Globally, the morbidity and mortality rate because of cancer is increasing constantly. In one estimate, approximately 1 688 780 new cases and 600 920 cancer deaths are expected to occur in 2017 in the United States alone.^1^ The modern era of cancer chemotherapy began in 1942; since then chemotherapeutic agents have been used extensively as single agents,^2^ in combination,^3^ and in conjunction with radiotherapy.^4-6^ However, development of resistance to chemotherapy^7-9^ and molecular-targeted therapies,^10-13^ and severe side effects^14^ are the major pitfalls of these agents. Therefore, life scientists are focusing more aggressively on the development of novel agents exhibiting antitumor and antimetastatic potential with no or minimal side effects.

The chemical compound or a substance produced by a living organism, found in nature is termed a “natural product.” Historically, chemical substances derived from animals, plants, and microorganisms have been found to have anticancer potential.^15,16^ Later, many compounds have been isolated from natural resources and among them, antitumor antibiotics are of great importance, including those isolated from various species of *Streptomyces*.^17-20^ Keeping in mind the role and contribution of natural products in drug discovery, one cannot ignore the therapeutic potential and contribution of edible and medicinal mushrooms in the field of medicine. Out of 14 000 different species of mushrooms, approximately 700 exhibit medicinal properties.^21^ Many of them have long been used in traditional Chinese medicine (TCM) and now interest has increased in the extraction of secondary metabolites for the discovery of novel drugs or lead compounds. Series of bioactive components^22-24^ have been isolated from mushrooms having antioxidant, antitumor, antiviral, antimicrobial and immunomodulating activities. Despite the discovery of many antitumor components of mushrooms, still, there is a need to increase the range of available compounds, find more effective and less toxic molecules and to understand deeply single and/or multiple targets, so as to battle cancer more precisely.

In this review, we have given evidence that the compounds of mushroom (Basidiomycota, Ascomycota) origin have antitumor and antimetastatic activity in in vitro and in vivo models, with a particular focus on the signaling pathways they target. Our first goal is to briefly portray a picture of genetic alterations associated with PI3K/AKT, Wnt-CTNNB1, andNF-κB signaling pathways and their role in the initiation and progression of cancer. Our second goal is to emphasize the antitumor potential of mushroom origin compounds in a signaling pathway-specific manner. It is our hope that appreciating the role of these mushroom originated compounds will help in targeting cancer in alteration-specific and cancer type–specific manner.

## The Anticancer Role of Mushrooms: Mechanistic Approach

In Asian countries, medicinal use of mushrooms has a long history; however, in recent decades, their use has also increased in other parts of the world. Many mushrooms are in clinical trials, and a few are used in clinics (Table 1). The tremendous therapeutic potential of edible and medicinal mushrooms is because of the lectin, arginine, ergosterol, β-glucan, and other bioactive substances in mushrooms. Some of the bioactive substances of mushroom origin, with experimental model type and possible molecular mechanisms are listed in Table 2. However, there are a few shortcomings in the already existing investigations, including limited studies on the interaction of mushroom components with different nodes of the signal transduction pathways. Few studies show the antitumor effects in vivo models and many lack the *IC*_50_ value of the agent under consideration. In order to target cancer more precisely, it is necessary to have a good understanding of both the molecular mechanisms underlying the development and progression of cancer and of the molecular targets of mushroom compounds at the same time. Furthermore, in this review, we discuss in some detail PI3K/AKT, Wnt-CTNNB1, and NF-κB signaling pathways, the occurrence of genetic alterations in them, the association of these genetic alterations in human cancers and how different nodes of these pathways are targeted by mushroom origin compounds.

**Table 1.** Mushrooms in Clinical Trials and in the Clinics.

Mushroom	Clinical Trial Description						
	Identifier	Investigator	First Received	Last Updated	Cancer/Condition	Phase	Status
*Maitake*	NCT02603016	Shunchang Jiao	2015	2015	Lung neoplasms	Phase 1	Ongoing
*Maitake*	NCT02603016	Shunchang Jiao	2015	2015	Breast carcinoma	Phase 2	Ongoing
*Reishi*	NCT02486796	Christina Shannon	2015	2016	Breast cancer, quality of life	Phases 1 and 2	Ongoing
*Agaricus blazei murill*	NCT00970021	Jon-Magnus Tangen	2009	2014	Multiple myeloma	Phase 2	Completed
*Coriolus versicolor*	NCT00680667	Carolyn Torkelson	2008	2012	Breast cancer	Phase 1	Completed
*Yunzhi*	NCT00647075	Pere Gascon	2008	2010	Breast cancer	Phase 4	Ongoing
*White button*	NCT00709020	Shiuan Chen	2008	2015	Breast cancer, cancer survivor	Phase 1	Completed
*White button*	NCT00779168	W. Twardowski	2008	2016	Prostate cancer	Phase 1	Ongoing
*Shiitake*	NCT00269555	Robert Hackman	2005	2010	Prostate cancer	Not mentioned	Completed
Mushroom	Clinical Description						
	Bioactive Substance		Commercial Name of Product		Mode of Action		Route of Administration
*Coriolus versicolor*	Protein bound polysaccharide		PSK, Krestin		Immunostimulant, inhibit tumor growth		Oral
*Coriolus versicolor*	Polysaccharide peptide		PSP		Immunostimulant, inhibit tumor growth		Oral
*Grifola frondosa*	Polysaccharide		Grifolan		Antiviral, antitumor, balance immune system		Oral
*Lentinula edodes*	Polysaccharide		Lentinan		Immunostimulant		Intravenously
*Schizophyllum commune*	Polysaccharide		SPG, Sonifilan		Immunostimulant		Intratumoral

**Table 2.** Effect of Mushroom’s Bioactive Substances on Different Cancer Types: In Vitro and In Vivo Experiments.^a^

Class	Mushroom Species/Reference	Bioactive Substance	Experimental Study		Concentration Used	IC_50_	Target/Mechanism
			Cancer Type	Model Type			
Edible mushrooms	*Albatrellus confluens* ^25^	Grifolin	Osteosarcoma	U2OS, MG63 cell line	0, 5, 10, 25, 50, or 100 μM	NG	Activation of caspase 3,9, cleavage of PARP, ⊗AKT, FOXO↓, GSK3↓
	*Albatrellus confluens* ^26^	Neogrifolin	Osteosarcoma	U2OS, MG63 cell line	0, 5, 10, 25, 50, or 100 μM	NG	Activation of caspase 3,9, cleavage of PARP, ⊗AKT, FOXO↓, GSK3↓, IAP↓
	*Auricularia auricula* ^27^	Polysaccharide	Liver cancer	HepG2, Bel-7404	0, 0.25, 0.5, and 1.0 mg/mL	NG	p27^kip^↑, cyclinD1/CDK4↓, cyclin E/CDK2↓, ⊗AKT at Thr 308 and Ser 473, pPTEN↑,Bcl-2 activation, Apoptosis, cCaspase 3 and 9↑
	*Grifola frondosa* ^28^	Polysaccharide	Liver cancer	Hep3B/Nude mice	0, 250, 500, and 1000 µg/mL, 10, 20 and 50 mg/kg	NG	PI3K ↓, cJun ↑, S phase arrest, apoptosis
	*Grifola frondosa* ^29^	Protein-bound polysaccharide	Breast cancer	MCF7 cell line	0, 18, 36, 91, 183, 367 mg/mL	NG	Apoptosis, BAK-1 ↑, cytochrome c transcript ↑
	*Lentinus crinitus* ^30^	Panepoxydone (PP)	Breast cancer	MDA-MB-453, MCF-7, MDA-MB-468 and MDA-MB-231 cell lines	2.5, 5, and 10 μM/2, 4, and 8 μM/3, 6, and 12 μM/7.5,15, and 30 μM	4, 5, 6, and 15 mM	Bax ↑, cPARP↑. Bcl-2↓, survivin↓. cyclin D1↓, caspase 3↓ FOXO1 ↓,⊗ IκBα
	*Lentinula edodes* ^31^	*Protein (Latcripin-1)	Lung cancer	A549 cell line	7.5, 15, and 30 µg/mL	NG	Apoptosis
	*Lentinula edodes* ^32^	*Protein (Latcripin-3)	Lung cancer	A549 cell line	7.5, 15, and 30 µg/mL	NG	Apoptosis/S phase arrest
	*Lentinula edodes* ^33^	*Protein (Latcripin-13)	Lung cancer	A549 cell line	6.25, 12.5, 25, 50, 100 and 200 µg/mL	NG	Apoptosis
	*Lentinula edodes* ^34^	*Protein (Latcripin-15)	Lung cancer	A549 cell line	12.5, 25, 50, 100, and 200 µg/mL	NG	Apoptosis
	*Lentinus edodes* ^35^	Ploysaccharide	Hepatocarcinoma of mouse	H22 cell line	0, 5, 50, and 500 µg/mL	NG	Apoptosis, G2/M Phase arrest
	*Pleurotus ferulae* ^36^	Terpenoids	Melanoma/Gastric cancer	B16F10,BGC823, GES-1 cell line/C57BL/6 female mice	0, 0.4, 0.8, and 1.6 mg/mL	NG	Apoptosis, G0/G_1_ arrest, MMP ↓, caspase 3↑
	*Pleurotus ostreatus* ^37^	Polysaccharide	Sarcoma	Sarcoma 180 cells /male BALB/c mice	100, 200, and 400 µg/mL and mg/kg	NG	TNF- and iNOS↑, phosphorylation of p65, IκB↓
	*Termitomyces clypeatus* ^38^	Sugar entities	Brain, breast, acute myeloid leukemia, lung, ovary, retinoblastoma	U373MG, MDA-MB-468, HepG2, HL-60, A549, U937, OAW-42 and Y-79/Male Swiss albino mice	0.01, 0.1, 1, 10, and 100 µg/mL; 200, 400 mg/kg	NG	Antioxidant/anticancer
Medicinal mushrooms	*Amauroderma rude* ^39^	Ergosterol	Breast cancer	MDA-MB-231, SK-BR-3, MDA-MB-468, MCF-7, 4T1	0, 50, 100, 150, 200, 250 and 300 µg/mL	NG	Growth inhibition and apoptosis. Fas↑, FasL↑, BimL↑, BimS ↑
	*Antrodia camphorata* ^40^	Ploysaccharide (ACE)	Hepatocellular carcinoma	HepG2 cell line	2.65, 5.3, 10.6, and 21.2 µg/mL	NG	Apoptosis
	*Antrodia camphorata* ^41^	Antroquinonol	Pancreatic carcinoma	PANC-1 and AsPC-1 cells	0.3, 1, 3, 10, and 30 μM	18.6 and 20.2 µM	⊗AKT at Ser 473, ⊗mTOR at Ser 2448
	*Antrodia camphorata* ^42^	Antroquinonol	Colon cancer	HCT15, HCT-116 and LoVo cells	0, 2.5, 5, 10, 20, 40, and 80 μM	34.8, >80, and 17.9 µM	PI3K/AKT/β-catenin signaling ↓
	*Antrodia camphorata* ^43^	4-Acetylantroquinonol B	Colorectal cancer	DLD-1, HCT-116, SW-480, RKO, HT-29	0, 10, 20, 30, 40, 50, and 60 μM	11.3, 34.6, 38.1, 22.3, 39.2 µM	Lgr5/Wnt/β-catenin, JAK–STAT↓
	*Cordyceps militaris* ^44^	Cordycepin		NRK-52E cell line	0, 0.5, 1, 2, and 5 µg/mL	NG	↓NF-κB
	*Fomes fomentarius* ^45^	Ploysaccharide (MFKF-AP1β)	Lung cancer	A549 cell line	25, 50, and 100 µg/mL	NG	Apoptosis
	*Grifola frondosa* ^46^	Sulfated polysaccharide	Liver cancer	HepG2	0, 10, 20, 40, 60, 80, and 100 µg/mL	61 µg/mL	Apoptosis, S phase arrest, notch1 ↓, IκB-α degradation, FLIP ↓, Caspase 3,8 ↑
	*Ganoderma lucidum* ^27^	Polysaccharide	Liver cancer	HepG2, Bel-7404	0, 0.25, 0.5, and 1.0 mg/mL	NG	p27^kip^↑, cyclinD1/CDK4↓, cyclin E/CDK2↓, ⊗AKT at Thr 308 and Ser 473, pPTEN↑, Bcl-2 activation, apoptosis, caspase 3 and 9↑
	*Inonotus obliquus* ^47^	Ergosterol peroxide	Colorectal cancer	HCT116, HT-29, SW620, DLD-1 CRC cell lines	0, 5, 10, and 20 µg/mL	NG	β-catenin pathway ↓
	*Phellinus linteus* ^48^	Protein-bound polysaccharide	Colon cancer	SW480 cell line	125, 250, 500, and 1000 μg/mL	NG	Wnt/β-catenin Pathway ↓, Cyclin D1↓, TCF/LEF↓
	*Phellinus linteus* ^49^	Hispolon	Human hepatoma cells	SK-Hep1 cells	4.6, 11.5, 22.8, 34.3, 45.7, 114, and 228 μM	NG	MMP2↓, MMP9↓, uPA ↓, ⊗ ERK1/2, ⊗PI3K/AKT, ⊗FAK
	*Phellinus linteus* ^27^	Polysaccharide	Liver cancer	HepG2, Bel-7404	0, 0.25, 0.5, and 1.0 mg/mL	NG	p27kip↑, cyclinD1/CDK4↓, cyclin E/CDK2↓, ⊗AKT at Thr 308 and Ser 473, pPTEN↑, Bcl-2 activation, apoptosis, caspase 3 and 9↑
	*Pleurotus pulmonarius* ^50^	Protein-bound polysaccharide	Liver cancer	Huh7, Hep3B, SMMC-7721, HepG2	0,25,50,100, 200, or 400 µg/mL	NG	VEGF-induced PI3K/AKT signaling↓

a *recombinant protein; ↑ upregulation; ↓ downregulation; ⊗ phosphorylation inhibition; NG not given.

## Targeting the PI3K/AKT Signaling Pathway in Cancer

Phosphoinositide 3-kinases (PI3Ks) are a key signal transduction system that on activation cause the activation of AKT and other downstream pathways.^51^ This signaling pathway has been reported to have a role in different cellular processes, including cell survival, proliferation, and differentiation.^52,53^ Several studies have declared that many of the components of this pathway are altered, including activation of growth factor receptor, *PIK3CA or AKT* amplification or mutation, and *PTEN* loss of function in common human cancers.^54-56^

Several lines of evidence indicate that mushroom compounds are capable enough of showing antitumor and antimetastatic activity by targeting various nodes of the PI3K/AKT pathway. In 2010, a group^49^ showed the dose-dependent inhibitory effect of “Hispolon” (*Phellinus linteus*) on invasion and motility of a highly metastatic liver cancer cell line (SK-Hep1), suggesting its role as an antimetastatic agent. According to their findings, this phenolic compound results in the down-regulation of MMP2, MMP9, and uPa. Together with this it also causes the phosphorylation inhibition of the ERK1/2, PI3K/AKT, and FAK pathways. In harmony with this study, the antiproliferative activity of proteoglycan (P1) from *Phellinus linteus* was demonstrated in multiple human cancer cells and in BALB/c-nu/nu mice bearing HT-29 cells.^57^ As far as the molecular mechanism is concerned, P1 resulted in a notable decrease in AKT, Reg IV, EGFR, and plasmatic PGE2 concentrations measured by ELISA. Similarly, Xu et al^50^ demonstrated the suppression of PI3K/AKT signaling in liver cancer cells by a polysaccharide-protein complex isolated from *Pleurotus pilmonarius* (PP). Their results indicated that PP not only significantly reduced in vitro cancer cell proliferation and invasion but also inhibited tumor growth in xenograft BALB/c nude mice when administered orally and intraperitoneally. As per their findings, these inhibitory effects are because of the inhibition of the autocrine VEGF-induced PI3K/AKT signaling pathway. In addition to this, when PP was used together with cisplatin, it resulted in the enhancement of liver cancer cell sensitization to cisplatin. It has been demonstrated before, that on treatment with cisplatin, BAD is phosphorylated at Ser 136 via AKT and this phosphorylation is needed for cell viability. When this AKT node was inhibited in ovarian cancer cells, they become sensitive to cisplatin.^58^ Most probably, the same mechanism might be involved in the case of liver cancer cells: On inhibition of PI3K/AKT signaling by PP, they become more sensitive to cisplatin.

Antroquinonol, a derivative of natural ubiquinone, has been reported to inhibit the phosphorylation of Akt and mTOR at Ser^473^ and Ser^2448^, respectively in human pancreatic carcinoma cells.^41^ This compound also resulted in the induction of cross-talk between apoptosis, autophagy, and senescence, explained by the upregulation of p21^Waf1/Cip1^ and K-ras.

In another study, polysaccharide-induced apoptosis was demonstrated in human hepatoma cells, and polysaccharide was extracted from 3 medicinal mushrooms *P linteus* (PL), *Ganoderma lucidum* (GL), and *Auricularia auricula* (AA).^27^ Three of the polysaccharides resulted in enhanced expression of p27^Kip^ in HepG2 cells and, on the other hand, suppressed the activity of cyclin D1/CDK4 and/or cyclin E/CDK2. AKT, one of the key nodes of PI3K/AKT pathway, once phosphorylated and activated results in the activation of downstream genes, that is, GSK3, FOXOs, thereby regulating key cellular processes involved in protein synthesis, cell survival, proliferation, and metabolism. However, both phosphorylation events (at Thr^308^ and Ser^473^) are required for full activation of AKT. Surprisingly, polysaccharides from these medicinal mushrooms suppressed AKT activity through the inhibition of AKT phosphorylation at Thr^308^ and/or Ser^473^. In addition, they also resulted in the activation of mitochondrial-mediated apoptosis and G_1_ and/or S phase arrest in HepG2 cells. After a few years, the same research group, in one of their recent studies^59^ on proteomic analysis of HepG2 cells treated with mushroom polysaccharide, revealed the differential expression of 59 proteins identified through MALDI-TOF-MS, where DJ-1 and 14-3-3 were the key proteins identified as a result of Protein-Protein interaction analysis. DJ-1 is a novel oncogene product that has been reported to negatively regulate the PTEN activity.^60^ Therefore, lower expression of DJ-1 can decrease the phosphorylation of AKT, whereas a higher expression of DJ-1 can increase AKT phosphorylation and cell survival.^61^ Interestingly, in this study, DJ- 1 protein was down-regulated in the treated HepG2 cells. One of the recent studies demonstrated the effect of antroquinonol (ANQ) on cancer stem cell-like properties in colon cancer.^42^ The authors demonstrated the interaction of ANQ with PI3K, identified by molecular docking, and further showed the suppression of PI3K/AKT/β-catenin signaling in colon cancer, which is crucial for regulating the expression of pluripotent genes.

The PI3K/AKT pathway has already been reported as one of the signaling pathways that are involved in acquiring chemotherapeutic drug resistance in cancer.^62^ The aforementioned studies suggest that compounds from mushrooms could lead to the restoration of drug sensitivity. Second, these compounds can not only be used as a single agent but may also be useful as adjuvant therapeutic agents in the treatment of cancer.

## Wnt/β-Catenin Pathway as a Therapeutic Target in Cancer

Wnts are the secreted glycoproteins that are involved in several critical cellular processes.^63^ They regulate many of the signaling pathways by β-catenin dependent or independent mechanisms.^64^ The occurrence of a high frequency of aberrations in the Wnt signaling pathway in many different cancers highlights the importance of this pathway to carcinogenesis. *APC, CTNNB1, AXIN1, FAM123B*, and *TCF7L2* are the key nodes of Wnt signaling that harbor somatic mutations associated with some of the common human cancers.^64^ The development and progression of sporadic colorectal cancer (CRC) and familial adenomatous polyposis (FAP)-associated disease are believed to be because of the mutations in *APC* (adenomatous polyposis coli) gene.^65^ In addition to this, two inactivating mutations often occur in the *APC* gene, one leading to allelic loss and other resulting in a truncated protein.^66,67^

Most mutations noted in the Wnt pathway results in the hyperactivation of this signaling pathway. Although mutations observed in genes that cause the inactivation of Wnt-CTNNB1 signaling has been identified in other disorders but not in cancer.^64^ Depending on the stage and type of cancer, Wnt-CTNNB1 signaling pathway can either promote or inhibit the tumor initiation, growth, metastasis and drug resistance. Herein, because of the limited scope of this review, we will only address the oncogenic role of Wnt-CTNNB1 signaling and its inhibition by different mushroom originated compounds.

As mentioned before mutated APC gene is common in colon cancer, so aberrant activation of Wnt-CTNNB1 signaling pathway results in its progression. Many researchers have attemptd to target cancer using mushroom components; one such study^48^ conducted on colon cancer in 2011 highlighted the antitumor property of polysaccharides extracted from *P linteus* (PL) mushroom. Researchers showed that this polysaccharide is capable of suppressing tumor growth, invasion and angiogenesis by down-regulating certain downstream genes (*cyclin D1, TCF/LEF*) of the Wnt signaling pathway in SW480 human colon cancer cells. In vivo anticancer potential of this mushroom extract was determined by the heterotrophic mouse model. A recent study done on colorectal cancer cell lines^47^ highlights the anti-proliferative activity of ergosterol peroxide isolated from Chaga mushroom *(Inonotus obliquus)*. According to the findings, ergosterol peroxide inhibited the nuclear β-catenin level, which resulted in the down-regulation of downstream genes. In another recent study,^43^ investigators claimed much better effect of 4-acetylantroquinonol (4-AAQB), closely related to a well-known antroquinonol, in suppressing colorectal cancer and cancer stem-like phenotype. As per their findings, 4-AAQB negatively regulates the genes responsible for the oncogenic effect and the signal transduction pathways that are involved in stemness.

Targeting Wnt- CTNNB-1 signaling by natural compounds is not only limited to colon cancer: the antitumor activity of the mushroom component against human meningioma cells has also been reported.^68^ Ganoderic acid A and ganoderic Acid DM suppress the expression of Wnt 5α/β and β-catenin and also cause the phosphorylation of GSK3β, which results in the proteaosomal degradation of β-catenin in the cytoplasm. On reduced β–catenin activity expression of β-catenin target genes (*c-myc, cyclin D1*, and *VEGF*) also reduces, thus giving anti-tumorigenic effect in meningioma cells. Das et al have also included normal human neuron cells in their study and fortunately, all the compounds, including ganoderic acid A and ganoderic acid DM have no toxic effects on normal cells. These compounds might, therefore, be potential candidates for the pharmaceutical treatment of human meningiomas.

Hyperactivation of the Wnt-CTNNB1 pathway because of the mutations in *APC* and *AXIN1* limits the potential molecular targets for pathway modulation because factors acting upstream of the destruction complex are no longer necessary for the activation of a pathway. So, the prime focus of researchers is disrupting the interaction of CTNNB1 with TCF7L2, thus inhibiting the CTNNB1 transcription. In the above-given examples, on treatment with mushroom compounds, reduction in the CTNNB1 level and downstream genes is observed, indicating that mushroom extracts can be used to inhibit the CTNNB1-mediated gene transcription. However, targeting the Wnt-CTNNB1 pathway directly is not simple because of the redundancy of many components of the pathway, so further studies on the interaction of mushroom compounds and nodes of this pathway are needed to overcome the challenges in targeting Wnt-CTNNB1 signaling.

## Targeting the NF-κB Signaling Pathway in Cancer

The NF-κB signaling pathway is a signal transduction pathway that on activation controls the expression of hundreds of genes. In mammals, the NF-κB family consists of five related transcription factors, p50, p52, RelA (p65), c-Rel, and RelB.^69^ Activation of the NF-κB transcription factor may take place via the classical (canonical) pathway or through the alternative (noncanonical) pathway, where the common regulatory step is the activation of IκB kinase consisting of catalytic and regulatory subunits.^70,71^ NF-κB is well known for its role in immunity, inflammation, and oncogenesis.^72,73^ As far as the alteration in this signaling pathway is concerned, mutations in the core signaling components have been reported to have associations with human diseases.^74^

Numerous lines of evidence highlight the antitumor effect of edible and medicinal mushrooms through targeting the NF-κB signaling pathway. *Cordyceps militaris* is one of the traditional medicinal mushrooms, whose major bioactive component cordycepin (3-deoxyadenosine) has been reported to have both pro- and anti-apoptotic activity. In 2012, Kadomatsu et al^44^ investigated the effect of cordycepin on cell survival on exposure to TNF-α. They found that, on treatment with a sub-lethal dose of cordycepin, cells become sensitive to TNF-α mediated apoptosis, which takes place via suppression of pro-survival NF-κB. As far as the detailed molecular mechanism is concerned, mTORC1 activation by eIF2α was responsible for the suppression of NF-κB, a key pro-survival component in TNF-α signaling.

In another study, the molecular mechanism behind the antitumor effect of sulfated polysaccharide obtained from *Grifola frondosa* (S-GFB) on HepG2 was explored.^46^ The results indicated a typical change in cell morphology, S phase arrest, notch1 expression inhibition, degradation of IκB-α, translocation of NF-κB from the cytoplasm to nucleus and activation of caspase-3 and 8. In short, S-GFB resulted in apoptosis of HepG2 cells through the notch1/NF-B/p65-mediated caspase pathway.

Panepoxydone (PP), isolated from *Lentinus crinitus* was found to be a natural NF-κB inhibitor.^75^ Genetic alterations do exist in NF-κB signaling, and breast cancer is one of those cancers in which NF-κB is overexpressed, suggesting it as a therapeutic target. Arora *et al* isolated PP from *L crinitus* and reported it as an inhibitor of NF-κB. The antitumor effect of PP was reported in MCF-7 and triple negative breast cancer cell lines. According to their investigation, PP resulted in significant cytotoxicity, increased apoptosis, decreased invasion and migration in all tested cell lines. PP upregulated Bax and cleaved PARP, inhibited the phosphorylation of NF-κB with cytoplasmic accumulation and on other hand downregulated Bcl-2, survivin, cyclin D1, caspase-3, and FOXM1.^30^

Kong *et al* in 2014 ^37^ demonstrated the immunostimulating effect of polysaccharide (WPOP-N1) isolated from *Pleurotus ostreatus*, a famous edible mushroom. They highlighted the antitumor activity of (WPOP-N1) in an in vivo model and also suggested its role in the activation of macrophages through NF-κB signaling pathway.

## Conclusion

From the evidence presented in this review, we can anticipate the molecular mechanism of different mushroom originated compounds, where they exhibit anticancer potential through modulating different nodes of PI3K/Akt, Wnt-CTNNB1, and NF-κB signaling pathways and thus affecting a variety of cellular processes, for example, by induction of apoptosis, cell cycle arrest, increasing the antioxidant capacity of host, and interfering with tumor angiogenesis. Because a plethora of genes/proteins associated with signal transduction pathways are deregulated during the development and progression of cancer, they may thus serve as targets to different components of mushrooms depending on their location. As indicated, some of the mushroom compounds target a single node of a signaling pathway and some have multiple targets in the same and/or different signaling pathway. This property highlights the vertical or horizontal blockade of the signal transduction pathways in cancer by these compounds. In addition, studies presented here also highlight the role of mushroom components in restoring drug sensitivity and their implications as single and adjuvant therapeutic agents. However, a choice of which mushroom compounds to select for the treatment of cancer depends on multiple factors and needs further investigation.

Extensive studies have demonstrated the anticancer potential of different mushrooms in preclinical models however, they still have significant limitations. In many of the studies, the anticancer potential of mushroom extracts has been demonstrated instead of specific active components, and where the action of specific active component(s) originated from mushroom has been reported, it lacks the interaction of mushroom components with the reported nodes (proteins/enzymes) of the signaling pathways. Most of the investigators have not determined the IC_50_ values of the bioactive component used against cancer, and many preclinical studies are in their initial phases. Future studies are needed to explore the structure of the bioactive components, their interaction with different nodes of signaling pathways, and their preclinical effects on more complicated animal models that can closely mimic the human tumors in terms of genetic profile and tumor microenvironment.

This review provides the possible molecular insight of the action of mushroom origin compounds and it would be helpful in designing anticancer arsenals from mushroom for combating cancer.
